# Association Between Z Drugs Use and Risk of Cognitive Impairment in Middle-Aged and Older Patients With Chronic Insomnia

**DOI:** 10.3389/fnhum.2021.775144

**Published:** 2021-12-09

**Authors:** Fang Guo, Li Yi, Wei Zhang, Zhi-Jie Bian, Yong-Bo Zhang

**Affiliations:** Department of Neurology, Beijing Friendship Hospital, Capital Medical University, Beijing, China

**Keywords:** benzodiazepines, Z drugs, cognitive impairment, chronic insomnia, elderly

## Abstract

**Background:** Benzodiazepines (BZDs) and Non-BZDs (NBZDs) have been widely used for patients with chronic insomnia. Long-term uses of BZDs may cause cognitive impairment and increase the risk for dementia in older patients. NBZD as an agonist of the GABA_A_ receptor complex includes eszopiclone, zopiclone, zolpidem, and zaleplon, also collectively known as Z drugs. However, evaluations for an association between cognitive impairment and Z drug use have been limitedly performed. This study aimed to investigate the association between the risk of cognitive decline and exposure to Z drugs in middle-aged and older patients with chronic insomnia.

**Methods:** Investigations were performed on patients with chronic insomnia who visited the outpatient Department of Neurology, Beijing Friendship Hospital, and were assessed for the global cognitive function (MoCA) and memory (AVLT), executive function (TMT-B), visuospatial ability (CDT), verbal function (BNT-30), and attention (DST). Multiple regression analysis was conducted to determine the independent factors of cognition and evaluated the effect of Z drug use (zolpidem and zopiclone) on cognition.

**Results:** A total of 120 subjects were identified. In our analysis, BZD exposure density (*P* = 0.025, OR = 1.43, 95% CI, 1.25–1.86) was an independent risk factor of cognitive impairment in middle-aged and older patients with chronic insomnia. Neither Z drug use (*P* = 0.103) nor Z drug exposure density (*P* = 0.765) correlated with global cognitive function. Moreover, there was a positive association between Z drug use and attention [(*P* = 0.002, OR = 0.42, 95% CI, 0.24–0.73)]. Additionally, income level (*P* = 0.001, OR = 0.23, 95% CI, 0.10–0.53), severity of insomnia (*P* = 0.019, OR = 1.20, 95% CI, 1.03–1.40) and age (*P* = 0.044, OR = 1.07, 95% CI, 1.00–1.14) were also independent factors of global cognitive function.

**Conclusion:** BZD exposure density was an independent risk factor of cognitive impairment in middle-aged and older patients with chronic insomnia, but no correlation was found between Z drug use and cognitive impairment. Moreover, the use of Z drugs seemed to be associated with protection for attention. The use for prescription of BZDs, in this case, should be avoided or limited to low doses. Due to the addiction and tolerance, Z drugs should also be prescribed with great caution in middle-aged and elderly patients.

## Background

In recent years, with the increasing competition and the quickening pace of modern life, the incidence rate of insomnia has been drastically increasing, which seriously affects people’s overall health. Insomnia is presented in around 10–30% of the global population, and about 50% of which suffer from chronic insomnia (Bhaskar et al., 2016). It is reported that in healthy subjects, sleep disorders increase levels of beta-amyloid (Aβ) in the cerebrospinal fluid (CSF), leading to the gradual progression toward neurodegeneration and the emergence of mild cognitive impairment (MCI) (Livingston et al., 2017; Atkin et al., 2018; Burke et al., 2018). Animal studies have shown that sleep restriction increases the susceptibility to aβ aggregation-induced memory impairment in mice, accompanied by the increase of plasma corticosterone and pro-inflammatory cytokines in the brain, resulting in memory impairment and synaptic damage (Kincheski et al., 2017). Therefore, sleep disorders have become the major risk factor for the development of Alzheimer’s disease (AD) (Hennawy et al., 2019). Treatment of sleep disorders may reduce the risk of probable future AD (Burke et al., 2019).

The treatment of chronic insomnia mainly includes cognitive behavioral therapy (CBT), light therapy, complementary and alternative drug therapy. Although CBT is the recommended first-line treatment (Riemann et al., 2017), there are several limitations in its clinical implementation. Therefore, benzodiazepines (BZDs) and their receptor agonist Z drugs are commonly used for insomnia. However, long-term use of BZDs in the elderly causes serious side effects, which not only lead to drug dependence and tolerance but also increase the risks of fall and fractures (Dyer et al., 2020). Moreover, the effects of BZDs on cognition in the elderly are highly debated. Some studies have reported that long-term use may even increase the risk of permanent cognitive impairment (Billioti et al., 2014; Shash et al., 2016; Chan et al., 2017), while others have shown the contrary results (Imfeld et al., 2015; Bietry et al., 2017; Desmidt et al., 2019; Grossi et al., 2019; Dyer et al., 2020). Although the exact pharmacological association between BZDs and cognitive decline remains uncertain, it was determined that the use of BZDs in the elderly should be avoided or limited to short-term therapy.

Z drugs as an agonist of the BZD receptor component of the GABA_A_ receptor complex are commonly used for insomnia. Z drugs usually include Zolpidem, Zopiclone, Eszopiclone, and Zaleplon. There are limited clinical studies on the association between Z drugs and cognitive impairment. Cheng et al. (2017) have reported that the use of a high cumulative dose of zolpidem increases the risk of AD among the elderly living in Taiwan. Shih et al. (2015) have suggested that the effect of zolpidem on cognition in patients with AD remains uncertain. Furthermore, the association between the Z drugs’ use and the risk of developing cognitive impairment in middle-aged and older patients with chronic insomnia is unknown. The aim of this study was to evaluate the effect of Z drugs on cognition among middle-aged and older patients with chronic insomnia.

## Materials and Methods

### Subjects

This case-control study was conducted from March 2019 to April 2021. Patients with chronic insomnia were recruited from the outpatient care at the Department of Neurology, Beijing Friendship Hospital affiliated with Capital Medical University. The study was approved by the Ethics Review Committee of Beijing Friendship Hospital Affiliated to Capital Medical University (2019-P2-051-01). The inclusion criteria for the subjects were: (1) Patients with chronic insomnia who met the Diagnostic and Statistical Manual of Mental Disorders, 5th edition (DSM-V) diagnostic criteria and had the Pittsburgh Sleep Quality Index (PSQI) score > 7; (2) age range 50–80 year; (3) patients who had been educated for more than 6 years and could complete the cognitive function test and other tests specified in the program; (4) patients signed informed consent. While the exclusion criteria included: (1) Patients having secondary insomnia caused by physical diseases (such as sleep apnea syndrome, restless legs syndrome, periodic physical activity, rapid eye movement sleep disorder, etc.); (2) patients with schizophrenia, bipolar disorder, delirium, and other mental disorders who meet DSM-IV diagnostic criteria; (3) alcohol and drug dependence or abuse; (4) patients with fatal or unstable organic diseases (including serious heart, liver and kidney diseases, tumors, etc.); (5) Hamilton Depression Scale (HAMD) (17 items) score > 17, Hamilton Anxiety Scale (HAMA) score > 14. (6). Patients with dementia at baseline (such as AD, Parkinson’s disease, Lewy body dementia, frontotemporal dementia); (7) patients with a history of stroke, symptoms and signs of any neurological deficit, and responsible lesion; (8) patients with brain trauma, epilepsy, encephalitis, hydrocephalus, brain tumors and other neurological diseases leading to cognitive impairment; (9) patients having abnormal laboratory indexes: moderate anemia, fasting blood glucose more than two times of the upper limit of normal, creatinine level more than 1.5 times of the upper limit of normal, liver enzymes (aspartate aminotransferase or alanine aminotransferase levels) more than two times of the upper limit of normal; (10) jet lag or work shift-induced chronic insomnia; (11) systolic blood pressure > 180 mmHg or < 90 mmHg, and diastolic blood pressure > 120 mmHg or < 60 mmHg; and (12) patients receiving medications for improving cognition, including donepezil, galanthamine, kabbalatin, memantine, escitalopram oxalate, citalopram hydrobromide, sertraline hydrochloride, fluoxetine hydrochloride, fluvoxamine maleate, trazodone hydrochloride, mirtazapine, or potential drugs with negative impacts on cognition, including any of anticholinergics, antipsychotics, antiepileptics, or glucocorticoids.

### Assessment of Cognition and Insomnia

Based on the diagnostic parameters, patients were divided into normal cognition and MCI groups. The MCI group met the definition of the International Working Group (IWG) on MCI, such as (1) patients’ or insiders’ report, or experienced clinicians detected cognitive impairment; (2) objective evidence of impairment of one or more cognitive domains (from cognitive tests); (3) complex instrumental daily living ability might be slightly damaged, but retained independent daily living ability; (4) patients didn’t meet the diagnostic criteria for dementia (Winblad et al., 2004). In this study, patients who met the (1), (3), (4) of the criterion of IWG on MCI, and following conditions: (1) MoCA score ≤ 13 (illiterate group), ≤ 19 (primary school group), ≤ 24 (junior middle school and above group), (2) MMSE scale > 17 (illiterate group), > 20 (primary school group), > 24 (junior middle school and above group, were defined as MCI patients (Lu et al., 2011). Chinese Version of Mini-Mental State Examination (MMSE) (Li et al., 2016), Chinese Version of Auditory Verbal Learning Test (AVLT) (Zhao et al., 2012), Chinese Version of Trail Making Test-B (TMT-B) (Tombaugh, 2004), Chinese Version of Boston Naming Test-30 (BNT-30) (Williams et al., 1989), Chinese Version of Clock Drawing Test (CDT) (Chan et al., 2005), and Chinese Version of Digit Span Test (DST) (Groth-Marnat and Baker, 2003) were used to assess the memory, executive function, verbal function, visuospatial ability and attention. Chinese Version of the International Physical Activity Questionnaire (IPAQ) (Lou and He, 2019) was used to assess the daily exercise. We used the Chinese Version of PSQI scale (Zhang et al., 2016) to evaluate the severity of insomnia. Age at onset of insomnia (years), duration of insomnia (years), use of sedative-hypnotics, and family history of insomnia were also recorded for the analysis.

### Benzodiazepines and Z Drugs Use

Categorization was recognized according to the World Health Organization’s Anatomical Therapeutic Chemical classification system. Estazolam, lorazepam, diazepam, and clonazepam were categorized as BZDs. Zolpidem and zopiclone were BZD-related drugs or Z drugs. Duration of drugs used (years), frequency of drug use (times/week), BZDs and Z drugs use, BZDs’ and Z drugs’ exposure density (mg/d) were recorded. The first two indexes were obtained from patients, and the latter two were calculated from prescription registered data 1 year before the recruitment. The exposure density of each BZD or Z drug, which corresponded to an average 1-day exposure, equaled the total doses in milligrams divided by the duration of use in days. Each drug use and exposure density were calculated separately, and the overlapping drugs’ use were calculated, respectively, according to each drug.

### Confounders

We recorded known potential confounders for cognitive impairment, including marital status, living alone, income level, education level, and insomnia, and comorbidities, including hypertension, diabetes mellitus, coronary heart disease, and dyslipidemia. Patients were divided into two groups based on their annual income, such as the low-income group [annual income ≤ 50,000 Renminbi (RMB)] and the non-low-income group (annual income > 50,000 RMB). We used the years of education to show the education level. Depression is an important mental differential diagnosis of cognitive impairment. HAMD-17 items and HAMA scales were used to evaluate depression and anxiety disorder. Daily physical activity was assessed by International Physical Activity Questionnaires (IPAQ) (short format). The collected laboratory indexes included fasting blood glucose (mmol/l), triglyceride (mmol/l), total cholesterol (mmol/l), low-density lipoprotein cholesterol (LDL-C, mmol/l), high-density lipoprotein cholesterol (HDL-C, mmol/l), serum uric acid (μmol/l) and serum albumin (g/l) levels.

### Statistical Analysis

All statistical analyses were conducted using Statistics Package for Social Science (SPSS), version 22.0. Continuous variables were expressed as mean ± standard deviation (SD) or median (inter-quartile range) according to the data distribution. Categorical variables were expressed as numbers and percentages. The independent sample *t*-test or Mann-Whitney U test was performed to compare continuous variables. The chi-squared (χ^2^) test or Fisher exact test was performed for categorical variables. Correlation analysis was used to analyze the associations between cognition and drug use or other social-demographic, clinical, and laboratory variables. Multiple logistic regression analysis was conducted to determine the independent factors of cognition. Statistical significance was defined by *P*-value < 0.05 (two-tailed).

## Results

### Social-Demographic and Clinical Characteristics of Patients

Among the 120 patients, the mean age of the patients with chronic insomnia was 60.38 ± 6.60 years (range, 50–77 years), with 34 (38.30%) men. The average age at onset of insomnia and duration of insomnia were 47.45 ± 13.18 years (range, 16–73 years) and 12.89 ± 12.84 (range, 0.5–50 years), respectively. One hundred and thirteen (94.17%) patients had difficulty in falling asleep, 88 (73.33%) had difficulty in maintaining sleep, 30% woke up early, and most patients had two or three types of sleep disorders. Sixty-four (53.33%) patients had taken BZDs or Z-drugs for their insomnia. The average duration and frequency of sedative-hypnotics were 8.92 ± 12.39 years and 5.80 ± 2.07 times a week, respectively. BZDs were used by 25.00% (*n* = 30), while 35.00% used Z drugs (*n* = 42). The reported BZDs were estazolam (*n* = 14), lorazepam (*n* = 8), diazepam (*n* = 6), and clonazepam (*n* = 2), the Z drugs used included zolpidem (*n* = 23) and zopiclone (*n* = 18). Their average exposure densities were 0.57 ± 1.04 mg/d for BZDs and 4.47 ± 4.50 mg/d for Z drugs, respectively. Fifty-six (46.67%) patients used BZD or Z drug alone, while eight (6.67%) patients used BZD combined with Z drug. The concomitant illness of hypertension, diabetes mellitus, coronary heart disease, dyslipidemia, and gout was 31.67, 11.67, 6.67, 35.00, and 5.83%, respectively.

### Correlations Between Global Cognitive Function, Social-Demographic and Clinical Characteristics

The 120 patients were divided into the normal cognition group (*n* = 62) and MCI group (*n* = 58). Table 1 shows the socio-demographic and clinical characteristics and the comparison between the two groups. Compared to the MCI group, patients with normal cognition had younger age (*P* = 0.003), better income level (*P* = 0.000), a higher education level (*P* = 0.000), and lower PSQI scores (*P* = 0.019). However, there were no significant differences in terms of gender, BMI, marital status, living with others, HAMA and HAMD scores, age with the insomnia onset, insomnia duration, concomitant illness, laboratory indexes, and daily exercise.

**TABLE 1:** Patient characteristics in normal cognition and cognitive impairment groups.

	Patients with normal cognition (*n* = 62)	Patients with cognitive impairment (*n* = 58)	Univariate *(P)*
Male	13 (20.97%)	21 (36.21%)	0.064
Age (years)	58.00 (54.75, 63.25)	61.00 (57.00, 67.00)	0.003
Married/cohabitating	61 (98.39%)	53 (91.38%)	0.180
Living with others	61 (98.39%)	54 (93.10%)	0.322
Low income	16 (25.81%)	37 (63.79%)	0.000
Education (years)	13.00 (11.75, 15.00)	11.00 (9.00, 12.00)	0.000
BMI, kg/m^2^	24.09 ± 3.20	23.81 ± 2.60	0.603
Smoking, %	12 (19.35%)	9 (15.52%)	0.580
**Concomitant illness**			
Hypertension, %	18 (29.03%)	20 (34.48%)	0.521
Diabetes mellitus, %	5 (4.17%)	9 (16.67%)	0.204
Coronary heart disease, %	2 (3.23%)	6 (10.34%)	0.232
Dyslipidemia, %	20 (32.26%)	22 (37.93%)	0.515
Gout, %	3 (4.84%)	4 (6.90%)	0.928
**Insomnia**			
Age at onset (years)	50.00 (40.00, 56.50)	50.00 (42.25, 59.00)	0.793
Insomnia duration (years)	7.00 (3.75, 15.25)	10.50 (3.00, 20.50)	0.211
PSQI scores	13.69 ± 3.23	14.90 ± 2.24	0.019
Family history of insomnia, %	14 (22.58%)	17 (29.31%)	0.338
**Laboratory indexes**			
Fasting blood glucose, mmol/l	5.54 ± 0.32	5.31 ± 0.53	0.213
Triglyceride, mmol/l	1.02 ± 0.12	1.26 ± 0.28	0.452
Total cholesterol, mmol/l	4.13 ± 0.78	4.75 ± 0.86	0.072
LDL-C, mmol/l	2.25 ± 0.65	2.74 ± 0.59	0.085
HDL-C, mmol/l	1.45 ± 0.16	1.60 ± 0.27	0.236
Serum uric acid, umol/l	275.73 ± 47.85	289.76 ± 43.52	0.560
Serum albumin, g/l	42.52 ± 2.90	41.77 ± 3.02	0.157
HAMD scores	6.50 (4.00, 11.00)	8.00 (6.00, 10.00)	0.322
HAMA scores	7.00 (5.00, 12.25)	9.00 (6.75, 11.00)	0.149
IPAQ scores	924.00 (693.00, 2079.00)	1386.00 (693.00, 2845.00)	0.229

Correlation analysis showed that global cognitive function (MoCA score) was correlated with age (*r* = –0.25, *P* = 0.007), marital status (*r* = –0.22, *P* = 0.018), living with others (*r* = –0.20, *P* = 0.026), income level (*r* = 0.33, *P* = 0.000), education level (*r* = 0.48, *P* = 0.000), HAMD scores (*r* = –0.20, *P* = 0.033), history of diabetes mellitus (*r* = 0.21, *P* = 0.022) and coronary heart disease (*r* = 0.22, *P* = 0.018). Further, a multiple logistic regression analysis was performed to define the independent predictors of global cognitive function. Age (*P* = 0.044, odds ratio, OR = 1.07, 95% confidence interval (CI), 1.00–1.14), income level (*P* = 0.001, OR = 0.23, 95% CI, 0.10–0.53) and PSQI scores (*P* = 0.019, OR = 1.20, 95% CI, 1.03–1.40) were independently associated with global cognitive function (see Table 2).

**TABLE 2:** Multiple logistic regression analysis for factors independently associated with global cognition in insomnia patients.

	*P*-Value	Odds ratio	95% CI
Age (years)	0.044	1.07	1.00–1.41
Low income	0.001	0.23	0.10–0.53
PSQI scores	0.019	1.20	1.03–1.40

### Correlation Between Sedative-Hypnotics and Global Cognitive Function

For the BZD and Z drug users, Table 3 shows the comparison between normal cognition group (*n* = 20) and MCI group (*n* = 44). The patients with normal cognition had less BZD exposure density (*P* = 0.031), lower PSQI scores (*P* = 0.023), higher education level (*P* = 0.003), better income level (*P* = 0.000),and were mostly women (*P* = 0.024) with young age (*P* = 0.000). In multiple logistic regression analysis, there were four factors that independently predicted global cognitive function, including BZD exposure density (*P* = 0.025, OR = 1.43, 95% CI 1.25–1.86), PSQI scores (*P* = 0.023, OR = 3.58, 95% CI 1.19–10.70), income level (*P* = 0.022, OR = 0.01, 95% CI 0.11–0.40) and age (*P* = 0.013, OR = 1.61, 95% CI 1.11–2.33). The results indicated that patients with normal cognition were likely to have lower BZD exposure density. No significant correlation was found between global cognitive function and Z drug use, Z drug exposure density, duration and frequency of drugs used.

**TABLE 3:** Univariate analysis for factors associated with cognition in benzodiazepines and Z-drugs use patients.

	Patients with normal cognition (*n* = 20)	Patients with cognitive impairment (*n* = 44)	Univariate *(P)*
Benzodiazepine use	6 (30.00%)	24 (54.55%)	0.068
Benzodiazepine exposure density(mg/d)	0.00 (0.00, 0.50)	0.15 (0.00, 1.00)	0.031
Z drug use	16 (80.00%)	26 (59.09%)	0.103
Z drug exposure Density(mg/d)	4.29 (1.80, 5.00)	5.00 (0.00, 7.50)	0.765
Duration of drugs use (years)	2.00 (1.00, 10.00)	3.00 (1.00, 15.00)	0.290
Frequency of drugs use (times/week)	5.35 ± 1.82	6.00 ± 2.17	0.248
Male	4 (20.00%)	22 (50.00%)	0.024
Age (years)	57.20 ± 4.10	63.23 ± 6.56	0.000
Married/cohabitating	20 (100.00%)	34 (77.27%)	0.051
Living with others	20 (100.00%)	36 (81.82%)	0.103
Low income	2 (10.00%)	30 (68.18%)	0.000
Education (years)	15.00 (14.00, 16.00)	11.00 (9.00, 13.00)	0.003
BMI, kg/m^2^	22.54 ± 2.73	23.07 ± 2.51	0.454
Smoking, %	4 (20.00%)	10 (22.73%)	1.000
**Concomitant illness**			
Hypertension, %	8 (40.00%)	16 (36.36%)	0.781
Diabetes mellitus, %	2 (10.00%)	8 (18.18%)	0.642
Coronary heart disease, %	0 (0.00%)	6 (13.64%)	0.203
Dyslipidemia, %	10 (50.00%)	16 (36.36%)	0.303
Gout, %	0 (0.00%)	2 (4.55%)	0.846
**Insomnia**			
Age at onset (years)	49.00 (40.00, 54.00)	47.00 (30.00, 59.00)	1.000
Insomnia duration (years)	6.00 (5.00, 10.00)	13.50 (3.00, 28.00)	0.181
PSQI scores	12.90 ± 3.16	14.64 ± 2.58	0.023
Family history of insomnia,%	6 (30.00%)	18 (40.91%)	0.403
HAMD scores	11.00 (9.00, 12.00)	8.50 (6.00, 13.00)	0.116
HAMA scores	8.50 (7.00, 15.00)	10.00 (7.00, 12.00)	0.522
IPAQ scores	1697.00 (594.00, 2879.00)	2093.00 (532.00, 3102.00)	0.323

### Correlation Between Sedative-Hypnotics and Memory, Executive Function, Attention, Verbal Function, Visuospatial Ability

To better investigate the effect of sedative-hypnotics on cognition, the associations between sedative-hypnotics and five major cognitive domains were analyzed.

In the BZD users, there were lower MMSE scores (*P* = 0.013), MoCA scores (*P* = 0.003), and DST scores (*P* = 0.004) than the other patients. In addition, the patients were older (*P* = 0.000), with significant hypertension incidences (*P* = 0.003) associated with BZD exposure. In the Z drug users, patients exhibited higher MMSE scores (*P* = 0.003), MOCA scores (*P* = 0.029), long-term delayed recall of AVLT scores (*P* = 0.037), BNT-30 scores (*P* = 0.016), and DST scores (*P* = 0.002) compared with the patients without the use of Z drugs. In the univariate analysis, the HAMD scores (*P* = 0.020) were higher with Z drugs (see Tables 4, 5). In multiple logistic regression analysis, the BZD users had fewer DST scores (*P* = 0.017, OR = 2.02, 95% CI 1.14–3.58), while the Z drug users had higher DST scores (*P* = 0.002, OR = 0.42, 95% CI 0.24–0.73).

**TABLE 4:** Univariate analysis of the correlation between cognition and benzodiazepines.

	BZDs use (*n* = 30)	Non-BZDs use (*n* = 90)	*P*-value
MMSE scores	26.00 (23.00, 28.00)	28.00 (26.00, 28.25)	0.013
MoCA scores	21.20 ± 3.49	23.50 ± 3.65	0.003
Long-term delayed recall of AVLT scores	5.00 (4.00, 6.00)	7.00 (3.75, 9.75)	0.184
TMT-B scores	126.00 (81.00, 300.00)	85.00 (57.75, 217.00)	0.062
BNT-30 scores	25.80 ± 3.02	25.42 ± 3.30	0.581
CDT scores	4.00 (3.00, 4.00)	4.00 (3.00, 4.00)	0.596
DST scores	4.00 (3.00, 5.00)	5.00 (4.00, 5.25)	0.004
HAMD scores	10.00 (6.00, 13.00)	9.00 (6.75, 11.50)	0.766
HAMA scores	10.00 (6.00, 12.00)	9.00 (7.00, 15.25)	0.808
Male	12 (20.83%)	27 (20.83%)	0.311
Age (years)	64.20 ± 6.98	59.22 ± 5.52	0.000
Married/cohabitating	24 (80.00%)	85 (94.44%)	0.045
Living with others	24 (80.00%)	87 (96.67%)	0.009
Low income	18 (60.00%)	46 (51.11%)	0.398
Education (years)	11.27 ± 3.63	11.75 ± 3.37	0.505
BMI, kg/m^2^	22.95 ± 2.55	23.63 ± 2.86	0.247
Smoking, %	4 (13.33%)	19 (21.11%)	0.349
**Concomitant illness**			
Hypertension, %	16 (53.33%)	22 (24.44%)	0.003
Diabetes mellitus, %	6 (20.00%)	11 (12.22%)	0.450
Coronary heart disease, %	4 (13.33%)	8 (8.89%)	0.725
Dyslipidemia, %	8 (26.67%)	39 (43.33%)	0.105
Gout, %	2 (6.67%)	2 (2.22%)	0.557
**Insomnia**			
Age at onset (years)	50.00 (28.00, 58.00)	48.00 (42.25, 54.25)	0.872
Insomnia duration (years)	17.00 (5.00, 34.00)	10.00 (3.00, 15.25)	0.214
PSQI scores	14.27 ± 3.05	13.90 ± 2.71	0.535

**TABLE 5:** Univariate analysis of the correlation between cognition and Z drugs.

	Z drug use (*n* = 42)	Non-Z drug Use(*n* = 78)	*P*-value
MMSE scores	28.00 (25.75, 29.00)	26.00 (23.00, 27.00)	0.003
MoCA scores	24.00 (19.00, 26.25)	22.00 (17.00, 23.00)	0.029
Long-term delayed recall of AVLT scores	7.00 (3.75, 9.75)	5.00 (4.00, 5.00)	0.037
TMT-B scores	85.00 (59.50, 217.00)	126.00 (90.00, 300.00)	0.073
BNT-30 scores	26.48 ± 3.13	25.00 ± 3.18	0.016
CDT scores	4.00 (3.00, 4.00)	4.00 (3.00, 4.00)	0.413
DST scores	5.00 (4.00, 5.00)	4.00 (2.00, 5.00	0.002
HAMD scores	10.19 ± 4.36	8.23 ± 4.32	0.020
HAMA scores	11.00 (7.00, 15.25)	9.00 (5.00, 11.00)	0.094
Male	18 (42.86%)	21 (26.92%)	0.075
Age (years)	59.43 ± 5.31	61.03 ± 6.70	0.184
Married/cohabitating	38 (90.48%)	71 (91.03%)	1.000
Living with others	40 (95.24%)	71 (91.03%)	0.637
Low income	18 (42.86%)	46 (58.97%)	0.091
Education (years)	12.24 ± 3.25	11.30 ± 3.49	0.154
BMI, kg/m2	23.03 ± 2.86	23.69 ± 2.75	0.220
Smoking,%	12 (28.57%)	11 (14.10%)	0.055
**Concomitant illness**			
Hypertension, %	14 (33.33%)	24 (30.77%)	0.773
Diabetes mellitus, %	4 (9.52%)	13 (16.67%)	0.284
Coronary heart disease, %	4 (9.52%)	8 (10.26%)	1.000
Dyslipidemia, %	20 (47.62%)	27 (34.62%)	0.164
Gout, %	0 (0.00%)	4 (5.13%)	0.337
**Insomnia**			
Age at onset (years)	48.00 (38.75, 54.00)	54.00 (30.00, 69.00)	0.213
Insomnia duration (years)	10.00 (4.50, 17.75)	6.00 (2.00, 28.00)	0.755
PSQI scores	14.05 ± 2.73	13.96 ± 2.84	0.873

## Discussion

As far as we know, this was the first study to evaluate the effect of Z drugs on cognitive function in middle-aged and older patients with chronic insomnia. In all patients with chronic insomnia, we found that severity of insomnia, income level, and age were independent factors associated with global cognitive function (MoCA). Patients with higher PSQI scores and/or lower income levels were likely to have the worse global cognitive function. Among the sedative-hypnotics users, we observed BZD exposure density and PSQI score, income level, and age were independently associated with global cognitive function. It suggested that patients with normal cognition would have lower BZD exposure density, lower PSQI score, higher income level, and younger age. We found that neither Z drug use nor Z drug exposure density correlated with the global cognitive function. The use of BZDs might hurt attention, while the use of Z drugs might protect the verbal function at the same time.

The correlation of Z drugs and cognition in this study was not aligned with that of previous studies. Cheng et al. (2017) and Lee et al. (2018) have reported that the use of zolpidem is associated with an increased risk of AD among older people, especially a high cumulative dose, while Shih et al. (2015) has found that the effect of zolpidem on cognition in patients with AD remains uncertain. Our findings on BZD use were consistent with several previous studies that reported long-term use of BZDs increases the risk of cognitive decline (Billioti et al., 2014; Shash et al., 2016; Chan et al., 2017), though there are also some studies reporting no association between BZD use and cognitive impairment (Imfeld et al., 2015; Bietry et al., 2017; Desmidt et al., 2019; Grossi et al., 2019; Dyer et al., 2020). For retrospective studies, lacking well-controlling confounders, such as education level, marital status, depression, stroke, diabetes, hypertension, coronary heart disease, physical activity, smoking, medicines affected cognition, especially insomnia, might be the possible cause of these inconsistent findings. As known, insomnia increases the risk of developing AD. If we do not adjust the confounder of insomnia, the correlation between sedative-hypnotics and cognitive decline may not be reliable.

The mechanism of how sedative-hypnotics could increase the risk of cognitive decline is still unclear. There is a hypothesis that the cognitive reserve capacity of the elderly is limited after taking BZD for a long time. Because BZDs and Z drugs are positive regulators of the GABA_A_ receptor, they will reduce brain activation, decrease synaptic plasticity, and affect the ability of patients to create new memories (Stern, 2012). Secondly, the binding of BZDs to α5GABA_A_ subunit, which is mainly expressed in the hippocampus, impairs context memory information in monkey models. However, zolpidem does not impair the performance of visual cue-based tasks due to the affinity for α1GABA_A_ rather than α5GABA_A_ (Mohamad and Has, 2019). These results suggest that α5GABA_A_ receptor plays a special role in BZD-related cognitive impairment. It is thus speculated that BZD can increase the risk of cognitive impairment through α5GABA_A_ in the hippocampus, while Z drugs, which bind to α1GABA_A_ have a lower risk. However, another study has shown that the activation of α1GABA_A_ receptor can influence the spatial learning ability of rodents (Joksimovic et al., 2013). Our current study found no association between Z drugs and cognitive impairment, which could partly be explained by its affinity for α1GABA_A_ subunit rather than α5GABA_A_.

Another reason for this association might be related to the increase of slow-wave sleep. Insomnia increases neuronal activity, decreases the clearance of β-amyloid protein (A β) and tau, and increases the accumulation of Aβ plaque and tau protein by reducing slow-wave sleep, which leads to neurodegeneration and AD. Slow-wave sleep plays an important role in sleep-dependent declarative memory consolidation (Xie et al., 2013; Rasmussen et al., 2018). Insomnia patients with less slow-wave sleep show a decrease in declarative memory consolidation the next day (Lu and Goder, 2012). The Z drugs used in this study were zolpidem and zopiclone, which could maintain sleep stages and promote slow-wave sleep, which might be part of the reason why Z drugs did not lead to cognitive decline in this study. This might also be part of the reason why BZDs, which reduced slow-wave sleep impaired cognition. The molecular mechanisms of cognitive impairment caused by BZD and Z drug need a further prospective study to confirm.

In this study, the uses of BZDs and Z drugs in patients aged 50–80 years old with chronic insomnia were up to 53.33%. The high prevalence of drugs might vary due to doctors’ and patients’ ignorance of the risk of drug-induced falls, fractures, and cognitive decline, as well as the lack of attention to cognitive behavioral therapy. Besides, the exclusion of patients without sedative-hypnotics who had not visited the outpatient department might partly contribute to the high prevalence of drugs. Due to age-related pharmacokinetic and pharmacodynamic changes, the elderly were more sensitive to the effects of BZDs on the central nervous system (Trifiro and Spina, 2011). Therefore, the use of BZDs in this population might lead to daytime sedation and decreased alertness (Dell’Osso, 2013). Our study found that BZD exposure density was an independent factor of cognitive impairment in patients with chronic insomnia. This evidence indicated that the high dose use of BZDs should be avoided in middle-aged and elderly subjects. Like BZDs, Z drug may be problematic in those predisposed toward substance abuse (Krystal et al., 2019), this can induce addiction and tolerance. Furthermore, some Z drugs also have rebound insomnia and withdrawal effects. Therefore, Z drugs should also be prescribed with great caution and avoid abrupt cessation.

This study had several strengths. First, this study recorded the patients’ education and income level, marital status, smoking, physical activity, concomitant illness, laboratory examination, and sleep assessment so that well-controlled the confounders of cognition. Second, anxiety and depression are closely related to chronic insomnia and affect cognition. This study evaluated the degree of anxiety and depression of patients through HAMA and HAMD scales to avoid the interference of emotional disorders on cognition. Third, the medication of each patient was recorded in detail, including drug type, dose, medication time, and frequency, and provided data for clarifying the possible dose-effect relationship and the correlation between duration of exposure and cognitive decline. This study improved our understanding of the cognitive progression and emphasized the medication selection among patients with a high risk of developing dementia. Notably, the study design excluded patients taking antipsychotics and antidepressants, potentially avoiding the impact of the above drugs on cognition. Finally, this study assessed the cognitive status of patients in detail, including global cognitive function and memory, executive function, visuospatial ability, verbal function, and attention, to better evaluate the effect of BZDs and Z drugs on cognition. We were confident to the reliability of the conclusion of the correlation between hypnotics and cognition.

This study also has certain limitations. Insomnia is considered to be a prodromal symptom of dementia. The use of BZDs may not be a cause of dementia, but indeed insomnia is a clinical manifestation at an early stage of dementia. Our study did not completely exclude the possibility of reverse causation. The investigation was designed/performed as a single-center study with a limited sample size. We are recruiting the patients from multiple centers, where we can focus on increasing the enrollment for male gender. The electrophysiology data such as polysomnography (PSG), has been also included in our recent research. Since it is common in older adults, females, and people with medical and mental ill health to suffer from chronic insomnia (Taylor et al., 2007; Schutte-Rodin et al., 2008), male patients accounted for 38.30% in this study. A larger sample prospective study is being proposed to confirm the findings and extend our research.

## Conclusion

We found that BZD exposure density was an independent risk factor of cognitive impairment in middle-aged and older patients with chronic insomnia, but no correlation was found between Z drug use and cognitive impairment. Moreover, our study showed that Z drug use might protect attention compared with non-Z drug use. Additionally, income level, the severity of insomnia, and age were also independent factors of global cognitive function. Our findings suggested that the cognitive status should be extensively evaluated and monitored in middle-aged and older patients with sedative-hypnotics; the prescription of BZDs should be avoided or limited in low doses. Although there is no evidence of cognitive decline in this study, the occurrence of side effects and death of Z drug may be similar to that of BZDs (Gunja, 2013). Z drugs should also be prescribed with great caution in middle-aged and elderly.

## Data Availability Statement

The raw data supporting the conclusions of this article will be made available by the authors, without undue reservation.

## Ethics Statement

The studies involving human participants were reviewed and approved by the Ethics Review Committee of Beijing Friendship Hospital Affiliated to Capital Medical University (2019-P2-051-01). The patients/participants provided their written informed consent to participate in the study.

## Author Contributions

LY and WZ participated in data collection. FG participated in data analysis, prepared and revised the manuscript, and created tables. Y-BZ oversaw data analysis, manuscript revision, and financial support. All authors read and approved the manuscript.

## Conflict of Interest

The authors declare that the research was conducted in the absence of any commercial or financial relationships that could be construed as a potential conflict of interest.

## Publisher’s Note

All claims expressed in this article are solely those of the authors and do not necessarily represent those of their affiliated organizations, or those of the publisher, the editors and the reviewers. Any product that may be evaluated in this article, or claim that may be made by its manufacturer, is not guaranteed or endorsed by the publisher.

## Abbreviations

BZDs: Benzodiazepines
MMSE: Mini-mental State Examination
MoCA: Montreal Cognitive Assessment
AVLT: Auditory Verbal Learning Test
TMT-B: Trail Making Test-B
BNT-30: Boston Naming Test-30
CDT: Clock Drawing Test
DST: Digit Span Test
CSF: Cerebrospinal Fluid
AD: Alzheimer’s Disease
DSM-V: Diagnostic and Statistical Manual of Mental Disorders, 5th edition
PSQI: Pittsburgh Sleep Quality Index
HAMD: Hamilton Depression Scale
HAMA: Hamilton Anxiety Scale
IPAQ: International Physical Activity Questionnaires
LDL-C: Low-density Lipoprotein Cholesterol
HDL-C: High-density Lipoprotein Cholesterol.
